# Supplementary material for: Treatment and Prognosis of Myocardial Infarction Outside Cardiology Departments
Source: J Clin Med. 2020 Dec 30;10(1):106. doi: 10.3390/jcm10010106 (PMC7795967; doi:10.3390/jcm10010106)
Supplement: Supplementary file 1 [file jcm-10-00106-s001.pdf]

Journal of
Clinical Medicine

MDPI

Supplementary Materials

Table S1. Variables entered in the logistic regression analysis of predictors for CD care.

Variable	B	S.E.	Wald	df	Sig.	Exp(B)
Age_years	0.071	0.017	17.408	1	0.000	1.074 (1.039–1.111)
Male sex, (y/n)	−0.228	0.310	0.539	1	0.463	0.797 (0.434–1.462)
Active smoker, (y/n)	0.797	0.498	2.560	1	0.110	2.219 (0.836–5.888)
Chest pain, (y/n)	−1.922	0.316	36.935	1	0.000	0.146 (0.079–0.272)
Atrial fibrillation, (y/n)	0.576	0.375	2.368	1	0.124	1.780 (0.854–3.708)
History of PCI, (y/n)	−0.654	0.410	2.541	1	0.111	0.520 (0.233–1.162)
History of major bleeding, (y/n)	0.987	1.019	0.937	1	0.333	2.682 (0.364–19.780)
History of TIA or stroke, (y/n)	0.276	0.392	0.493	1	0.483	1.317 (0.610–2.842)
Heart failure, (y/n)	−0.410	0.400	1.052	1	0.305	0.664 (0.303–1.453)
ST elevation MI, (y/n)	−1.806	0.526	11.764	1	0.001	0.164 (0.059–0.461)
Chronic kidney disease, (y/n)	−0.124	0.631	0.039	1	0.844	0.883 (0.256–3.043)
COPD, (y/n)	0.323	0.445	0.526	1	0.468	1.381 (0.578–3.300)
Metastatic cancer, (y/n)	2.892	0.888	10.618	1	0.001	18.035 (3.166–102.725)
Dementia, (y/n)	0.459	0.543	0.714	1	0.398	1.582 (0.546–4.588)
CRP at admission, mg/l	0.007	0.003	4.613	1	0.032	1.007 (1.001–1.013)
Hemoglobin at admission, (g/L)	−0.013	0.008	2.718	1	0.099	0.987 (0.972–1.002)
Creatinine at admission, µmol/L	0.004	0.003	1.675	1	0.196	1.004 (0.998–1.010)
Oxygen saturation, %	−0.029	0.023	1.574	1	0.210	0.971 (0.928–1.016)
Systolic blood pressure, mmHg	−0.003	0.006	0.297	1	0.586	0.997 (0.986–1.008)
Heart rate, bpm	−0.003	0.007	0.180	1	0.671	0.997 (0.983–1.011)
RLS >1, (y/n)	1.914	0.706	7.341	1	0.007	6.780 (1.698–27.077)
Constant	−1.583	3.086	0.263	1	0.608	0.205

PCI – percutaneous coronary intervention. TIA – transient ischemic attack. COPD – chronic obstructive pulmonary disease. CRP – C-reactive protein. RLS – reaction level scale.

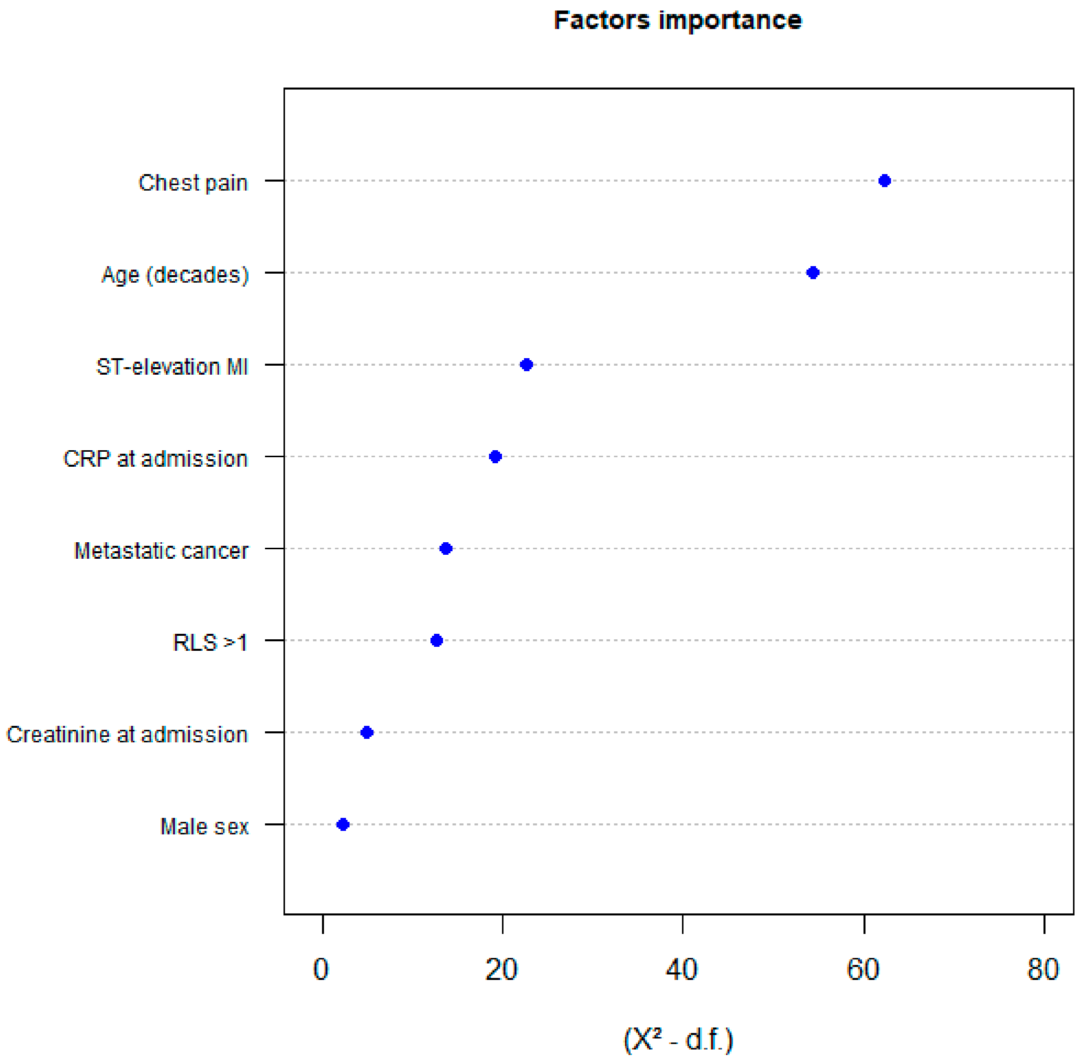

Figure S1. Factors importance in affecting the probability for CD care. MI – myocardial infarction. CRP – C-reactive protein. RLS – reaction level scale.

Table S2. Investigations, treatment and follow up stratified by MI type.

	**Cardiology Department**		**Other Department**		***p* for**	
	**Type 1**	**Type 2**	**Type 1**	**Type 2**	**Type 1**	**Type 2**
In-hospital, total n	798	170	78	75		
Treatment, n (%)						
Fondaparinux	398 (49.9)	90 (52.9)	24 (30.8)	9 (12.0)	0.001	<0.001
Low molecular weight heparin	41 (5.1)	18 (10.6)	12 (15.4)	20 (26.7)	<0.001	0.001
Antibiotics	78 (9.8)	53 (31.2)	21 (26.9)	46 (61.3)	<0.001	<0.001
Investigations, n (%)						
Echocardiography	559 (70.1)	79 (46.5)	9 (11.5)	26 (34.7)	<0.001	0.085
Coronary angiography	635 (79.6)	64 (37.6)	6 (7.7)	3 (4.0)	<0,001	<0.001
PCI (% of all patients)	538 (67.4)	26 (15.3)	4 (5.1)	0 (0.0)	<0.001	<0.001
Discharge, total n	744	165	60	56		
Medications, n (%)						
RAAS blockers	585 (78.6)	106 (64.2)	29 (48.3)	31 (55.4)	<0.001	0.237
Acetylsalicylic acid	721 (96.9)	131 (79.4)	51 (85.0)	41 (73.2)	<0.001	0.336

Other platelet inhibitors	661 (88.8)	80 (48.5)	30 (50.0)	15 (26.8)	<0.001	0.005
Beta blockers	662 (89.0)	136 (82.4)	45 (75.0)	48 (85.7)	0.001	0.569
Statins	642 (86.3)	94 (57.0)	23 (38.3)	16 (28.6)	<0.001	<0.001
Anticoagulants	50 (6.7)	25 (15.2)	4 (6.7)	11 (19.6)	0.987	0.432
Scheduled follow up, n (%)						
Specialist	600 (80.6)	96 (56.5)	7 (11.7)	17 (22.7)	<0.001	<0.001
Primary health care	101 (14.7)	50 (29.4)	38 (63.3)	22 (29.3)	<0.001	0.990
No follow up	21 (2.8)	19 (11.2)	15 (25.0)	29 (38.7)	<0.001	<0.001

PCI – percutaneous coronary intervention, RAAS – Renin angiotensin aldosterone system.

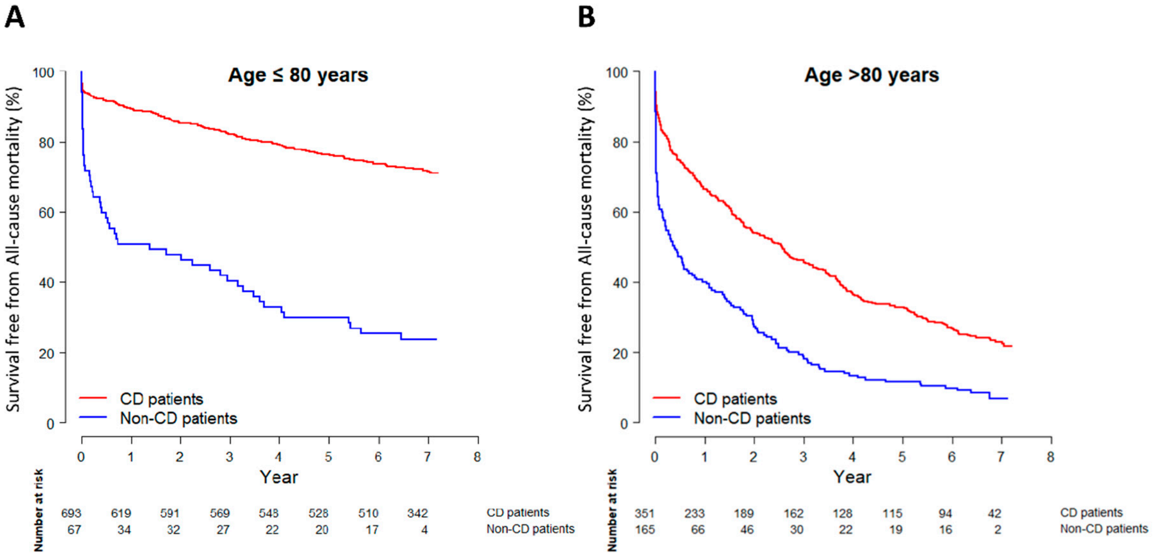

Figure S2. Crude total follow-up survival curves for patients treated at a cardiology department (red) and patients treated outside a cardiology department (blue). A) Patients ≤ 80 years. B) Patients > 80 years.

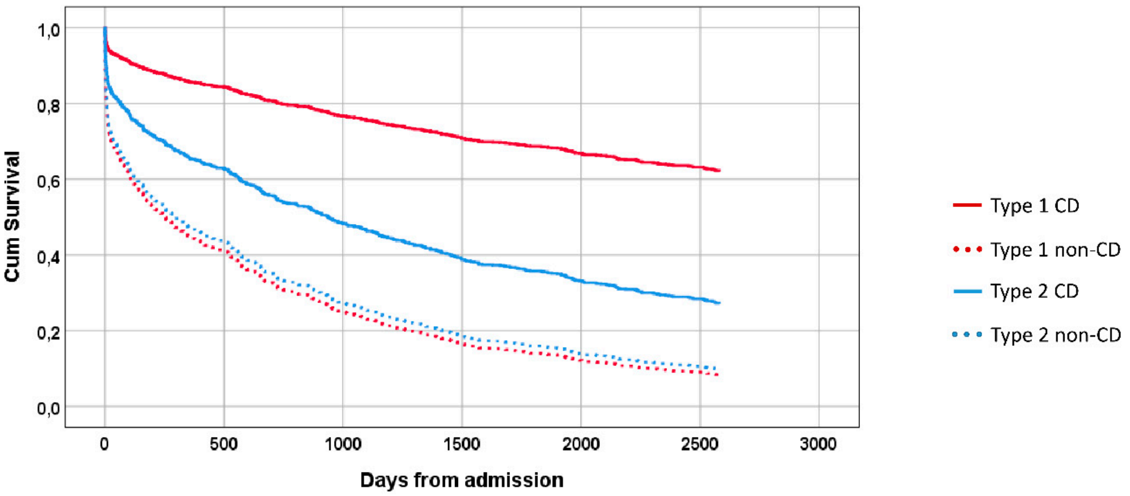

Figure S3. Crude total follow-up survival curves for type 1 MI (red) and type 2 MI patients (blue) stratified by cardiology department (solid) or non-cardiology department (dotted) care.
